# Autonomic Dysfunction in Mild Cognitive Impairment: Evidence from Power Spectral Analysis of Heart Rate Variability in a Cross-Sectional Case-Control Study

**DOI:** 10.1371/journal.pone.0096656

**Published:** 2014-05-06

**Authors:** Paola Nicolini, Michele M. Ciulla, Gabriella Malfatto, Carlo Abbate, Daniela Mari, Paolo D. Rossi, Emanuela Pettenuzzo, Fabio Magrini, Dario Consonni, Federico Lombardi

**Affiliations:** 1 Geriatric Unit, Fondazione IRCCS Ca’ Granda Ospedale Maggiore Policlinico, Department of Clinical and Community Sciences, University of Milan, Milan, Italy; 2 Cardiovascular Diseases Unit, Fondazione IRCCS Ca’ Granda Ospedale Maggiore Policlinico, Department of Clinical and Community Sciences, University of Milan, Milan, Italy; 3 Department of Cardiology, Ospedale San Luca, Istituto Auxologico Italiano IRCCS, Milan, Italy; 4 Epidemiology Unit, Fondazione IRCCS Ca' Granda Ospedale Maggiore Policlinico, Milan, Italy; University of Adelaide, Australia

## Abstract

**Background:**

Mild cognitive impairment (MCI) is set to become a major health problem with the exponential ageing of the world's population. The association between MCI and autonomic dysfunction, supported by indirect evidence and rich with clinical implications in terms of progression to dementia and increased risk of mortality and falls, has never been specifically demonstrated.

**Aim:**

To conduct a comprehensive assessment of autonomic function in subjects with MCI by means of power spectral analysis (PSA) of heart rate variability (HRV) at rest and during provocative manoeuvres.

**Methods:**

This cross-sectional study involved 80 older outpatients (aged ≥65) consecutively referred to a geriatric unit and diagnosed with MCI or normal cognition (controls) based on neuropsychological testing. PSA was performed on 5-minute electrocardiographic recordings under three conditions—supine rest with free breathing (baseline), supine rest with paced breathing at 12 breaths/minute (parasympathetic stimulation), and active standing (orthosympathetic stimulation)—with particular focus on the changes from baseline to stimulation of indices of sympathovagal balance: normalized low frequency (LFn) and high frequency (HFn) powers and the LF/HF ratio. Blood pressure (BP) was measured at baseline and during standing. Given its exploratory nature in a clinical population the study included subjects on medications with a potential to affect HRV.

**Results:**

There were no significant differences in HRV indices between the two groups at baseline. MCI subjects exhibited smaller physiological changes in all three HRV indices during active standing, consistently with a dysfunction of the orthosympathetic system. Systolic BP after 10 minutes of standing was lower in MCI subjects, suggesting dysautonomia-related orthostatic BP dysregulation.

**Conclusions:**

Our study is novel in providing evidence of autonomic dysfunction in MCI. This is associated with orthostatic BP dysregulation and the ongoing follow-up of the study population will determine its prognostic relevance as a predictor of adverse health outcomes.

## Introduction

Mild cognitive impairment (MCI) is a clinical entity that lies along the continuum from cognitively normal ageing to dementia [1]–[3]. It is characterized by a slight cognitive impairment, greater than expected for an individual's age and education but not severe enough to warrant a diagnosis of dementia, that does not substantially interfere with functional independence, although there may be some minimal deficits in the more complex instrumental activities of daily living [1]–[3]. It stands out as a major public health problem because it is increasingly recognized to be the prodromal stage of dementia, with an annual conversion rate ranging from 5 to 15% across different studies [3] and it affects a consistent portion of the population. In fact the prevalence of MCI among older adults aged 65 and over has been reported to be between 11 and 17%, i.e. from two to four times that of dementia [4], [5], and the absolute number of subjects with MCI is bound to rise exponentially with the rapid ageing of the population. Indeed in Italy, the third oldest country in the world with its 20% prevalence of elderly [6], the number of older adults is estimated to soar from just above 12 million in 2010 to just above 21 million in 2043 [7].

Heart rate variability (HRV) is the physiological phenomenon by which the heart rate (HR), far from displaying metronome-like regularity, changes from beat to beat, producing fluctuations in the time intervals between consecutive R waves (RR intervals) on an electrocardiographic (ECG) recording. HRV is considered to reflect the influence of the two limbs of the autonomic nervous system (ANS) –orthosympathetic and parasympathetic- on sinus node activity [8]–[10]. The analysis of HRV, available in most commercial Holter devices, thus provides a simple, non-invasive and reliable method for the assessment of autonomic function and as such has been extensively employed in clinical research [11].

In the literature there are two main lines of indirect evidence that suggest an association between MCI and autonomic dysfunction. First, MCI is considered a condition of predementia and a number of studies have demonstrated an impaired autonomic function in dementia, whether by clinical autonomic tests [12]–[16] or HRV analysis [17]–[21]. Also, a recent study has shown that, within a group of patients with Alzheimer's disease (AD), there was a positive correlation between cognitive status and parasympathetic HRV indices [22]. Second, MCI is characterized by a slight cognitive impairment and several studies, although with some exception [23], have revealed a significant association between the performance on cognitive tests and HRV-derived autonomic function across different age groups and conditions. In fact a relationship between cognitive functioning and HRV has been found in infants [24], children [25], young [26]–[29], middle-aged [29], [30] and older adults [31], [32] as well as in subjects suffering from anxiety [33]–[35]. To the best of our knowledge there are only two studies that have addressed the issue of autonomic function in MCI subjects relative to cognitively normal controls. The first [36] is a transcranial Doppler study, primarily conceived to investigate cerebrovascular reactivity to vasodilator/vasoconstrictor stimuli, that only collaterally reports a deficient response of the HR to hypercapnia in MCI patients. The second is a study by Zulli et al. [19] who evaluate autonomic function by means of HRV analysis, but fail to highlight differences in HRV indices between MCI and controls, possibly due to the lack of fully-controlled conditions (24-hour out-of-hospital ECG with power spectral analysis (PSA) performed on a 1-hour period during which the patient was instructed to remain supine and awake) as well as of provocative tests that may unmask a more a subtle dysautonomia by challenging the ANS [37].

The potential clinical significance of autonomic dysfunction in MCI is manifold. Reduced HRV has been found to be associated with increased all-cause mortality in the general elderly population [11] and in MCI subjects the relative risk of mortality, compared to controls, has indeed been reported to vary from 1.7 to 2.3 [38], [39]. Autonomic dysfunction can lead to blood pressure (BP) dysregulation [40] which can in turn prompt syncope and falls via hypotensive episodes [41] and potentially also through white matter lesions (WMLs) [42], [43] that impair balance and gait [44]. In fact the autonomic symptom score was documented to be a predictor of falls in dementia [45]. Falls are an important cause of morbidity, mortality and institutionalization in elderly people [46], and there is emerging data on MCI as a risk factor for falls [47], [48]. Moreover, particularly relevant to MCI is the hypothesis that BP dysregulation may result in cerebral hypoperfusion and brain damage that contribute to the progression of cognitive decline towards overt dementia. Support to such hypothesis comes from different works in the literature. There is a wealth of studies, in elderly subjects spanning a wide range of cognitive states, that report the association of BP dysregulation –in terms of absolute BP values, circadian BP pattern, orthostatic BP changes and BP variability (BPV) – with both cognitive impairment [19], [49]–[52] and WMLs/silent cerebral infarcts [42], [43]. Also, in older adults there is consistent evidence in favour of the role of silent cerebrovascular damage in predicting the likelihood and rate of subsequent cognitive decline and the risk of conversion to dementia [53], and within a group of MCI subjects it has been shown that reduced HRV indices correlate with increasing WML severity [54].

Lastly, autonomic dysfunction can be affected by a broad spectrum of medications - not only cardioactive ones [11]- and this is a matter of general concern in elderly subjects in whom polypharmacy is common [55] as well as a question of specific interest in MCI patients to whom cholinesterase inhibitors may be prescribed on the basis of their risk of progression [3].

The aim of this study was to evaluate autonomic function in subjects with MCI, compared with cognitively normal controls, by means of PSA of HRV applied to a controlled experimental protocol including two provocative tests: active standing (orthosympathetic stimulation) and paced breathing at 12 breaths/min (parasympathetic stimulation).

## Methods

### Study population

**Figure 1:**
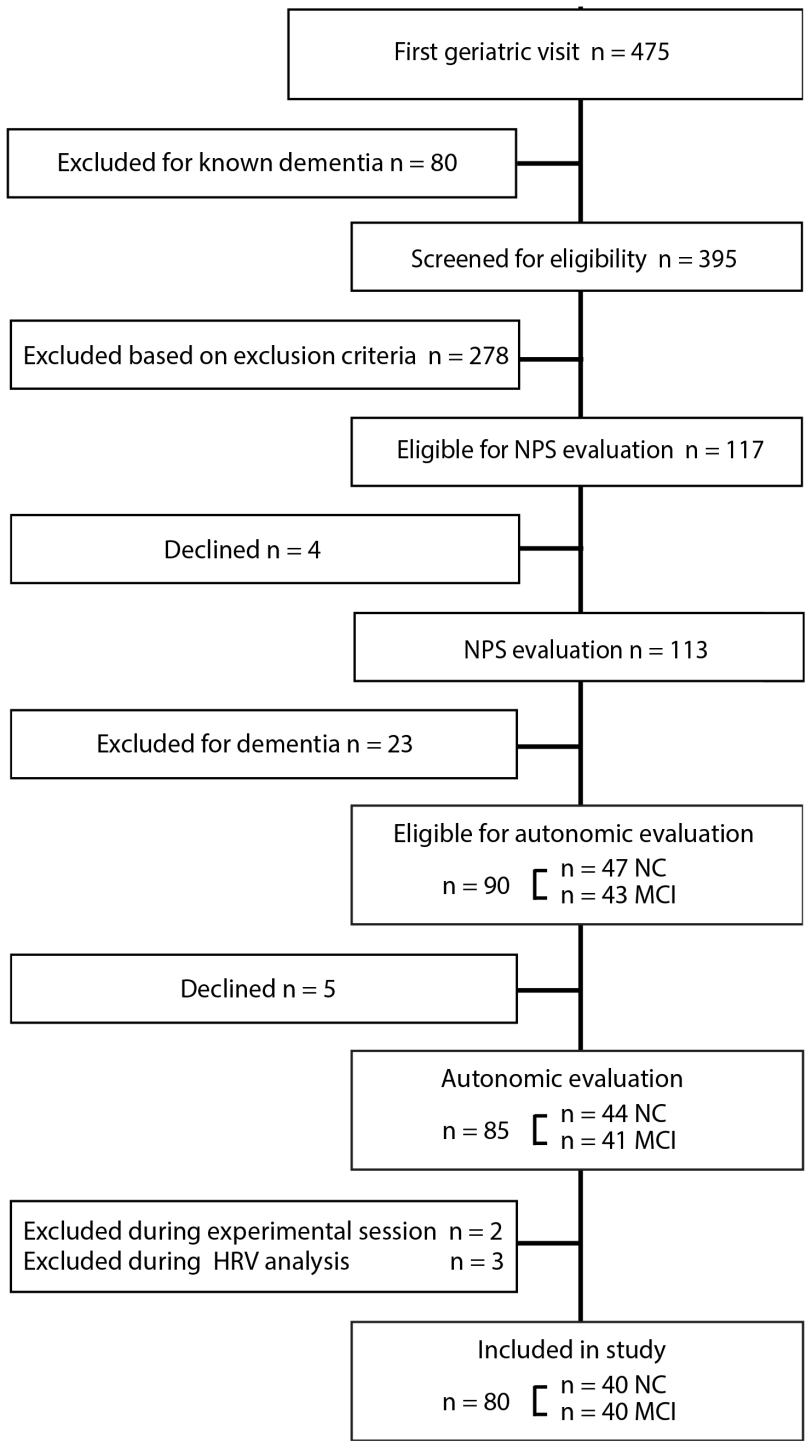
Flow diagram of the study population. NPS: neuropsychological; NC: normal cognition (controls); MCI: mild cognitive impairment; HRV: heart rate variability.

In this cross-sectional case-control study we considered for inclusion 475 community-dwelling older subjects (aged ≥65) who consecutively attended a first geriatric visit at the Geriatric Outpatient Unit of the Fondazione IRCCS Ca’ Granda Ospedale Maggiore Policlinico in Milan, Italy, from January to December 2012, to which they had been referred by their general practitioners (GPs) for a broad spectrum of age-related health problems. 80 subjects with a known diagnosis of dementia were excluded while the remaining 395 were assessed for eligibility by applying a number of exclusion criteria (see later). We thus identified 117 eligible subjects who were all invited to undergo an on-site neuropsychological evaluation. Of the 113 subjects who agreed to neuropsychological testing, 23 were diagnosed with dementia and were excluded, leaving 90 subjects with a diagnosis of MCI or normal cognition (NC). Of these, 5 declined participation so that the study eventually enrolled 85 subjects (n = 41 MCI: cases, n = 44 NC: controls), all of them recruited from the same population (Figure 1). Since all eligible subjects were invited to undergo neuropsychological testing independently of cognitive functioning there was no selection of the sample in such sense.

Exclusion criteria were: 1) non-sinus rhythm (atrial fibrillation and other arrhythmias, paced rhythms) since HRV analysis is, by definition, performed on sinus beats; 2) clinical conditions with an established and significant effect on HRV: history of heart disease (coronary artery disease (CAD), heart failure (HF)) [8], diabetes mellitus [8], neurological and psychiatric diseases (Parkinson's disease, stroke, major depression) [8], [56], [57], and severe diseases (respiratory, renal, hepatic, autoimmune and neoplastic) [58]; 3) use of several cardioactive medications: beta-blockers, alpha-blockers, centrally-acting calcium-channel blockers (CCBs), class I and III antiarrhytmic drugs, digoxin; 4) use of several psychotropic medications: tricyclic antidepressants, selective serotonin-noradrenaline reuptake inhibitors (SNRIs), atypical antidepressants, antipsychotics and cholinesterase inhibitors.

Among cardioactive medications we allowed diuretics, peripherally-acting CCBs and ACE-inhibitors (ACE-Is)/angiotensin II receptor blockers (ARBs) while among psychotropic medications selective serotonin reuptake inhibitors (SSRIs) and benzodiazepines were permitted. In particular, we recorded as benzodiazepine users subjects who were regular users, while intermittent users were asked to refrain from taking benzodiazepines in the two days prior to the neuropsychological and autonomic testing (48 hours corresponding to the maximum overall duration of action of long-half-life benzodiazepine diazepam) [59]. As far as the choice of medication exclusion/inclusion criteria is concerned please see the Discussion for details.

The diagnosis of MCI was made according to current consensus criteria [1], [2], [60]: objective cognitive impairment on neuropsychological testing, essentially preserved daily functioning and absence of dementia. The neuropsychological evaluation was based on a comprehensive battery of neuropsychological tests investigating different cognitive domains: 1) attention: digit cancellation [61] and bell [62] tests; 2) memory: prose recall [61], Rey-Osterrieth complex figure-delayed recall [63], digit span forward [64]; 3) executive functions: digit span backward [65], trail-making tests A and B [66], Weigl's colour-form sorting test [61], cognitive estimates [67], Raven's coloured progressive matrices [68], letter fluency [69]; 4) language: category fluency [61], picture naming [70], token test [61]; 5) visuospatial skills: Rey-Osterrieth complex figure-copy [63], copy of geometric figures [61]; 6) ideomotor praxis: De renzi's test [71]. MCI was diagnosed in individuals who were impaired in at least one cognitive domain. Impairment in a cognitive domain was defined as an abnormal performance in at least one test within that domain. Subjects were therefore classified as MCI if they showed an abnormal performance in at least one test of the neuropsychological battery. A test performance was considered abnormal when it fell below the 10 th percentile of published normative data for the Italian population [61]–[71], i.e. when it corresponded to the “worst” 10% of the distribution of scores obtained by available reference samples of cognitively normal subjects, age-, gender- and education adjusted where appropriate (please refer to Table S1 for the test scores and cut-offs). The 10 th percentile threshold was selected, in accordance with several authors (e.g [72]–[74]), because we believe that, relative to the other options of 1.5 and 1 standard deviations below norms (i.e. 7 th and 16 th percentile), also commonly accepted [75], it provides a more appropriate balance between the risk of under- and over-diagnosing MCI.

The 85 subjects taking part in the study underwent a clinical and autonomic assessment within one month from the neuropsychological evaluation.

### Clinical assessment

All subjects received a detailed clinical assessment to collect information on those characteristics that have been shown to affect HRV, either directly or via subclinical CAD [58], [76] and/or are relevant to a standard geriatric evaluation.

Sociodemographics (age, gender, education), lifestyle habits (smoking, coffee and alcohol consumption, physical activity), history of hypertension, medication history, family history of premature CAD [77] and routine blood tests (glucose, lipid panel) were recorded. Body mass index (BMI) was calculated as weight (kg)/height (m^2^); alcohol consumption was expressed as alcohol units/day (1 unit = 10 g alcohol) based on the average alcohol content by volume of the drinks consumed [78]; physical activity was quantified in MET (metabolic equivalent)-hours/week (1 MET = 1 kcal•kg^−1^•h^−1^) by means of a semistructured questionnaire in which MET values were assigned to different activities [79].

Target organ damage, in terms of left ventricular hypertrophy and carotid atherosclerosis, was also evaluated. Patients who did not have a recent (within the past year) echocardiogram or carotid Doppler scan underwent instrumental examination.

Functional status was assessed by means of the scales for the Basic Activities of Daily Living (BADL) [80] and for the Instrumental Activities of Daily Living (IADL) [81]; comorbidity by means of the Cumulative Illness Rating Scale (CIRS) severity (CIRS-s) and morbidity (CIRS-m) scores [82]; anxiety symptoms by means of the State Trait Personality Inventory-Trait anxiety subscale (STPI-T) [83]; depressive symptoms by means of the Geriatric Depression Scale short (15-item) version (GDS-s) [84]. The Mini Mental State Examination (MMSE) [85], corrected for age and education, was used to provide an estimate of global cognitive functioning, but, due to its recognized poor sensitivity to early cognitive impairment [3], it was not included in the battery of diagnostic tests for MCI.

### Autonomic assessment

The autonomic assessment was carried out in a quiet room, with dimmed lighting and a comfortable temperature (22-24°C), between 8:30 and 11:30 a.m. in order to minimize the effect of circadian changes in HRV [86]. Patients were instructed to consume a light breakfast and refrain from caffeinated beverages, alcohol, smoking and vigorous physical activity in the 12 hours prior to testing. After a standard 12-lead ECG, three-channel ECG recordings for HRV analysis were obtained by means of a digital Holter recorder (Spider View, Sorin Group Company).

**Figure 2:**
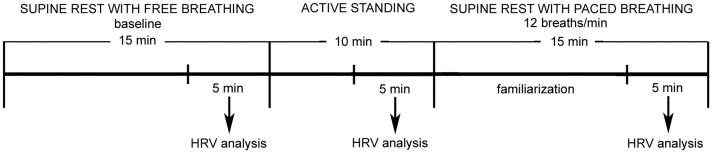
Timeline of the three stages of the experimental protocol. HRV: heart rate variability.

The protocol was composed of three stages (Figure 2): 1) supine rest with free breathing (baseline): 15 minutes during which the subjects were asked to remain awake, silent and still, breathing spontaneously. To allow for stabilization only the last 5 minutes were analysed; 2) active standing (orthosympathetic stimulation) [8]–[10]: 10 minutes during which the subjects were asked to remain still and silent, after standing upright in as smooth a motion as possible. We chose to analyse the last 5 minutes since 5 minutes is the time believed to be necessary for adjustment of autonomic function to posture [87] and lies at the upper range of the stabilization period adopted by most HRV studies involving orthostatic testing (e.g. [21], [22], [88]). We opted for an active rather than passive standing test because the former is more easily implemented in routine geriatric clinical practice and there is evidence that the two manoeuvres are comparable in terms of changes in autonomic balance [89]; 3) supine paced breathing at 12 breaths/min (0.2 Hz) (parasympathetic stimulation) [10]: 15 minutes during which the subjects breathed, as regularly as possible and with a “comfortable” tidal volume, according to an electronic metronome set at 24 acoustic signals per minute (2.5 s inspiration, 2.5 s expiration). Given the nature of our study population, this stage was made as simple as possible: the first 10 minutes were devoted to familiarization with the breathing protocol, without recording, and throughout the subjects were not asked to directly synchronize their breathing rhythm with the metronome but to follow voice indications from the experimenters. Within the 12–20 breaths/min respiratory rate range, associated with an enhancement of parasympathetic modulation [10], we chose the lowest respiratory rate because in a preliminary investigation we found it was the easiest for our subjects to maintain.

BP and HR were recorded at the end of the baseline period, after 1, 3, 5 and 10 minutes of active standing and at the end of the paced breathing, using a validated digital spyghmomanometer over the brachial artery (OMRON M6). Orthostatic hypotension (OH) was defined as a drop in systolic BP (SBP) ≥20 mm Hg and/or a drop in diastolic BP (DBP) ≥10 mm Hg [90] relative to baseline in at least one orthostatic recording; we extended the recording period beyond 3 minutes since milder autonomic dysfunction has been associated with delayed OH [91].

Since respiratory rate is a potent determinant of HRV [92], the spontaneous respiratory rate was visually assessed during the baseline and active standing periods. Subjects with a respiratory rate ≤9 breaths/min (≤ 0.15 Hz) were excluded due to the overlapping of the low- and high-frequency bands which precludes proper interpretation of PSA [93]. Based on the fact that emotional stress can have a confounding effect on HRV [8] and can be influenced by cognitive status, at the end of the active standing and paced breathing periods subjects were asked to rate their level of stress on a visual analogue scale (VAS) from 0 (no stress) to 100 (maximum stress).

### HRV analysis

HRV analysis was performed in the frequency domain (PSA) on each of three 5-minute ECG recordings (baseline, active standing, paced breathing), in accordance with the guidelines of the Task Force of the European Society of Cardiology and the North American Society of Pacing and Electrophysiology [8]. We used commercially available software (Synescope version 3.10, Sorin Group Company) which carries out PSA by means of the Fast Fourier Transform (FFT), after linear interpolation/resampling at 4 Hz of the discrete event series (to obtain a regularly time-sampled signal) and filtering with a Hanning window (to attenuate leakage effects). Although the software automatically detect non-sinus beats, the recordings were always manually overread by experienced investigators – blinded to the subjects' cognitive status- in order to ensure correct QRS complex classification and rhythm identification. The software corrects for ectopics by eliminating one RR interval before and after each non-sinus beat and replacing them with RR intervals computed by linear interpolation based on the surrounding normal beats [8]. This method is more appropriate than simple deletion (i.e. removal of the two non-normal RR intervals without replacement) because the latter leads to loss of data which may be critical in the case of short-term recordings and is also associated with a phase shift to which frequency-domain measures are particularly sensitive [94]. Since there is no clear indication in the literature as to the amount of ectopic beats that it is acceptable to remove or remove and interpolate [95], [96], with the exclusion threshold ranging from 1% [97] to 20% [98] of the total number of beats in different studies, we chose the most restrictive criterion. This is in line with the suggestion by Kamath et al. [95] that in a short-term study (less than 5 minutes) there should be no more than two to three ectopic beats. Thus, recordings with excessive supraventricular or ventricular ectopy- i.e. ectopic beats >1% of total beats - were excluded from analysis, as were those with other arrhythmias.

The resulting spectral indices were: 1) very low frequency power (VLF, ≤0.04 Hz), low frequency power (LF, 0.04-0.15 Hz), high frequency power (HF, 0.15–0.4 Hz) and total power (TP, ≤0.4 Hz) which are expressed in absolute units (ms^2^) and are computed by integrating the area under the power spectrum within the specified frequency range; 2) normalized LF (LFn) and high frequency (HFn) power which are expressed in normalized units (n.u.) and correspond to the absolute power divided by TP minus VLF and multiplied by 100; and 3) the LF/HF ratio which is the ratio of LF to HF. Two methodological issued deserve mention herein. First, we chose to mainly focus on those indices that better reflect the reciprocal interaction between the two branches of the ANS: LFn (an index of orthosympathetic modulation), HFn (an index of parasympathetic modulation) and the LF/HF ratio (an index of sympathovagal balance) [9].

TP, LF and HF were considered only for the sake of comparison with other studies because they suffer from some limitations. TP is an index of overall autonomic modulation and as such does not allow to separately assess the behaviour of the ortho- and parasympathetic limbs of the ANS. LF and HF are influenced by changes in TP in the same direction, which prevents appreciation of the fractional distribution of the energy (e.g. both decrease, albeit to different extents, during tilt) [8], [9]. Indeed the interpretation of the LF component is controversial: according to some investigators it is an index of predominant orthosympathetic modulation while others regard it as index of mixed ortho- and parasympathetic modulation [8]. Although HF is an index of predominant parasympathetic modulation [8], [9] there is evidence in the literature that HFn provides more accurate information on the state (activation/withdrawal) of the parasympathetic nervous system. In fact it has been shown that HFn, relative to HF, is characterized by a stronger (negative) correlation with the angle of tilt during graded orthostatic tilt [99] and with muscle sympathetic nerve activity quantified by microneurography during pharmacologically-induced BP changes [100]. VLF, whose physiological significance is still uncertain and probably related to the renin-angiotensin-aldosterone system, thermoregulation and vasomotor activity [9], was not taken into account.

Second, LFn, HFn and the LF/HF ratio were considered in each of the three stages of the protocol (baseline, active standing and paced breathing) but particular attention was devoted to their changes from baseline to stimulation : ΔLFn, ΔHFn, ΔLF/HF. These latter indices represent the response to the manoeuvre and are especially sensitive measures of autonomic modulation, extensively used in HRV studies (e.g. [88], [101]–[105]) because they explore the dynamic range of the ANS [37], [88].

### Statistical analysis

Data are reported as mean (standard deviation) for continuous variables and number (percentage) for categorical variables. The comparison between the two groups was carried out by means of: 1) the chi-squared test or Fisher's exact test for categorical variables; and 2) the independent samples Student's t-test or the Mann-Whitney U-test for continuous variables with a normal and non-normal distribution respectively; the normality of the data was assessed by using the Shapiro-Wilk test. In particular, HRV indices that were not normally distributed (HFn and LF/HF) were normalized by logarithmic transformation to base 10 (lg) in order to apply parametric statistics. Inflation of type I error due to multiple testing was controlled for by means of the Benjamini-Hochberg procedure [106] with the False Discovery Rate (FDR) set at the conventional level for alpha (q =  5%). This was preferred to the Bonferroni correction because the latter is known to be overly conservative in the case of highly correlated variables, like ours, leading to an undue loss in statistical power (i.e. an increase in false negatives) which would be inappropriate also given the exploratory nature of the study [107]. Multiple linear regression was used to adjust for potential confounders, which were chosen among the variables that were different between the two groups (i.e. CIRS and VAS-stress scores) and/or are particularly relevant to HRV or BP (i.e. medication use and HR). Specifically, the standing (and Δ standing) HRV indices were corrected for the CIRS-m and the standing VAS-stress scores, for medication use (ACE-Is/ARBs, diuretics, peripherally-acting CCBs, SSRIs, benzodiazepines) and for the average standing HR. Similarly, the paced breathing HRV indices were corrected for the CIRS-m and the paced breathing VAS-stress scores, for medication use and for the HR at the end of paced breathing. The SBP after 10 minutes of standing was corrected for the CIRS-m and VAS-stress standing scores as well as for antihypertensive medication use (ACE-Is/ARBs, diuretics, peripherally-acting CCBs). As far as comorbidity was concerned only the CIRS-m score was entered in the analyses to avoid redundancy (CIRS-m and CIRS-s being strongly correlated, Spearman's rho = 0.822, p<0.001). All variables satisfied the assumptions of normality (non-significance of the Shapiro-Wilk test), homoscedasticity (non-significance of Levene's test) and no-multicollinearity (variance inflation factor, VIF <5) [108]. A p value ≤0.05 was considered statistically significant. Analyses were performed by means of the statistical package SPSS version 19.0 for Windows (SPSS Inc., Chicago, IL).

### Ethics Statement

This study was conducted in accordance with the Declaration of Helsinki and was approved by the ethics committee of the Fondazione IRCCS Ca’ Granda Ospedale Maggiore Policlinico in Milan, Italy. All participants gave written informed consent to participation in the study.

## Results

Of the 85 participants, 5 were excluded during the experimental session (n = 1 baseline respiratory rate < 9 breaths/min, n = 1 postural vasovagal reaction) or the HRV analysis (n = 1 paroxysmal supraventricular tachycardia, n = 2 excessive ectopic beats), thus the study ultimately included 80 subjects, 40 with MCI and 40 with NC (control group) (Figure 1). It is important to note that, during paced breathing, all subjects breathed at the target respiratory rate of 12 breaths/min, as demonstrated by the centre frequency of the HF peak (0.2 Hz) on PSA.

The 80 subjects included were not significantly different from the 475 subjects considered for inclusion in terms of age (78.6 vs 78.5 years, p = 0.958), smoking habits (3.8% vs 4.6% smokers, p = 1.000) and use of most allowed medications (ACE-Is/ARBs 48.8% vs 46.1%, p = 0.661; peripherally-acting CCBs 13.8% vs 19.6%, p = 0.216; SSRIs 27.5% vs 31.2%, p = 0.511; benzodiazepines 18.8% vs 26.7%, p = 0.130). They were more frequently females (75.0% vs 55.2%, p = 0.001), had a borderline higher education (10.9 vs 10.0 years of schooling, p = 0.046), better functional status (BADL score 5.5 vs 5.2, p = 0.006; IADL score 7.1 vs 5.3, p<0.001), better cognitive functioning (MMSE score 27.7 vs 25.5, p<0.001), lesser comorbidity (CIRS-s score 1.5 vs 1.6, p<0.001; CIRS-m score 2.1 vs 2.5, p = 0.019), consumed a smaller number of medications (3.6 vs 5.3, p<0.001) and used less diuretics (20.0% vs 32.8%, p = 0.022); the prevalence of hypertension also tended to be lower in the study group, although the difference just failed to attain statistical significance (56.3% vs 67.2%, p = 0.057).

Table 1 shows the baseline characteristics of the study subjects. There were no significant differences between the two groups in most factors, including cardiovascular (CV) risk ones, potentially affecting autonomic function: sociodemographics, BMI, lifestyle habits, routine blood tests, prevalence of hypertension and target organ damage, family history of premature CAD, total number and main classes of medications, anxiety and depressive symptoms, BP values and respiratory rate (all p >0.1). In the MCI group, relative to controls, baseline HR was significantly higher (p = 0.035) and CIRS-s and CIRS-m scores were significantly lower (p = 0.029 and p = 0.006 respectively). As expected, MMSE and IADL scores were significantly lower in the MCI group (p<0.001 and p = 0.006 respectively). All subjects had normal blood levels of vitamin B12, folate and thyroid hormones. None had ischaemic ECG changes, pathological echocardiographic findings (except for slight valvular disease which is common in older people) or haemodinamically significant carotid stenosis. The neuropsychological test results are reported in Table S1 and indicate, as expected, a significantly poorer performance of MCI subjects on tests across all cognitive domains (all p<0.04).

**Table 1:** Baseline characteristics of the study subjects

Variable	NC (n = 40)	MCI (n = 40)	p
Age (years)	77.8 (4.5)	79.4 (5.3)	0.145 b
Gender, female	33 (82.5)	27 (67.5)	0.121 d
Education (years)	11.4 (4.4)	10.5 (4.4)	0.279 c
BMI (kg/m^2^)	24.1 (3.0)	24.6 (3.2)	0.431 b
Hypertension	21 (52.5)	24 (60.0)	0.499 d
SBP (mm Hg)	134.8 (19.2)	129.0 (17.4)	0.158 b
DBP (mm Hg)	75.2 (8.7)	73.6 (8.3)	0.404 b
Heart rate (beats/min)	65.2 (8.7)	69.6 (9.6)	0.035 b
Respiratory rate (cycles/min)	14.0 (2.4)	15.0 (2.8)	0.104 b
Smoking	2 (5.0)	1 (2.5)	1.000 e
Alcohol (AU/day)	1.3 (1.6)	1.3 (1.5)	0.968 c
Coffee (cups/day)	1.5 (1.0)	1.3 (1.1)	0.317 c
Physical activity (MET-hours/week)	68.4 (39.3)	57.6 (39.8)	0.144 c
Family history of premature CAD	7 (17.5)	7 (17.5)	1.000 d
Glucose (mg/dl)	87.7 (9.8)	91.5 (10.0)	0.107 b
Total cholesterol (mg/dl)	222.9 (37.8)	227.0 (33.9)	0.635 b
LDL cholesterol (mg/dl)	137.6 (33.0)	139.7 (28.0)	0.768 b
HDL cholesterol (mg/dl)	69.2 (20.5)	65.8 (18.2)	0.470 c
Triglycerides (mg/dl)	104.0 (32.4)	106.7 (36.4)	0.745 b
Target organ damage			
LVH-echocardiography	16 (40.0)	20 (50.0)	0.369 d
Carotid atherosclerosis			
Thickening (IMT >0.9 mm)	35 (87.5)	30 (75.0)	0.152 d
plaque/s	33 (82.5)	29 (72.5)	0.284 d
Number of medications	3.3 (1.7)	3.8 (2.2)	0.311 b
Antihypertensive medications			
ACE-Is/ARBs	16 (40.0)	23 (57.5)	0.117 d
Diuretics	7 (17.5)	9 (22.5)	0.576 d
CCBs (peripherally-acting)	3 (7.5)	8 (20.0)	0.105 d
Psychotropic medications			
SSRIs	9 (22.5)	13 (32.5)	0.317 d
Benzodiazepines a	8 (20.0)	7 (17.5)	0.775 d
BADL score	5.5 (0.5)	5.6 (0.6)	0.325 c
IADL score	7.4 (1.2)	6.7 (1.6)	0.006 c
MMSE score	28.6 (1.0)	26.8 (2.0)	<0.001 b
CIRS-s score	1.6 (0.2)	1.4 (0.2)	0.029 b
CIRS-m score	2.5 (1.3)	1.7 (1.1)	0.006 c
STPI-T score	19.5 (5.7)	19.6 (5.7)	0.843 c
GDS-s score	3.4 (3.1)	3.2 (2.8)	0.747 c

Continuous variables are expressed as mean (SD), categorical variables are expressed as n (%). Significant results are shown in bold typeface.

a Refers to regular use. Intermittent users (n = 2 in each group) were asked to refrain from use in the two days prior to testing;

b Student's t-test;

c Mann-Whitney's U-test;

d Chi-squared test;

e Fisher's exact test. NC: normal cognition (controls); MCI: mild cognitive impairment; BMI: body mass index; SBP: systolic blood pressure; DBP: diastolic blood pressure; AU: alcohol units (1 AU = 10 g of alcohol); MET: metabolic equivalent (energy expenditure index, 1 MET =  1 kcal•kg^−1^•h^−1^); CAD: coronary artery disease; LDL: low density lipoprotein; HDL: high density lipoprotein; LVH: left ventricular hypertrophy; IMT: intima-media thickness; ACE-Is: angiotensin converting enzyme inhibitors; ARBs: angiotensin II receptor blockers; CCBs: calcium-channel blockers; SSRIs: selective serotonin reuptake inhibitors; BADL: basic activities of daily living (score range 0–6, higher scores indicate greater functional independence); IADL: instrumental activities of daily living (score range 0–8, higher scores indicate greater functional independence); MMSE: mini mental state examination (score range 0–30, higher scores indicate better cognitive function); CIRS-s: cumulative illness rating scale severity (score range 1–5, higher scores indicate greater comorbidity); CIRS-m: cumulative illness rating scale morbidity (score range 0–13, higher scores indicate more severe comorbidity); STPI-T: state trait personality inventory- trait anxiety subscale (score range 10–40, higher scores indicate greater trait anxiety); GDS-s: geriatric depression scale short form (score range 0–15, higher scores indicate greater depressive symptoms).

Table 2 shows the characteristics of the subjects during the experimental session. After correction for multiple testing, the MCI group exhibited a significantly lower SBP after 10 minutes of standing (p = 0.047), a significantly higher HR at the end of paced breathing (p = 0.018), a significantly lower standing VAS-stress score (p = 0.041) and a marginally lower paced breathing VAS-stress score (p = 0.055). It should be remarked that the respiratory rate was comparable between the two groups, both in the standing position (p = 0.201) and in the change from baseline to standing (p = 0.526).

**Table 2:** Characteristics of the study subjects during the experimental session

Variable	NC (n = 40)	MCI (n = 40)	p	q ^d^
SBP after 1 min standing (mm Hg)	134.7 (20.3)	127.2 (18.0)	0.088 ^a^	0.157
DBP after 1 min standing (mm Hg)	82.1 (10.0)	78.1 (10.1)	0.080 ^a^	0.156
Heart rate after 1 min standing (beats/min)	71.6 (11.0)	75.7 (11.3)	0.115 ^b^	0.185
SBP after 3 min standing (mm Hg)	137.7 (18.9)	129.0 (16.7)	0.034 ^a^	0.092
DBP after 3 min standing (mm Hg)	82.1 (10.4)	78.7 (8.5)	0.111 ^a^	0.185
Heart rate after 3 min standing (beats/min)	68.8 (9.6)	73.3 (10.0)	0.047 ^a^	0.103
SBP after 5 min standing (mm Hg)	134.8 (20.5)	128.1 (16.6)	0.115 ^a^	0.185
DBP after 5 min standing (mm Hg)	81.7 (10.1)	78.6 (9.0)	0.150 ^a^	0.194
Heart rate after 5 min standing (beats/min)	69.5 (11.3)	73.6 (9.8)	0.085 ^a^	0.157
SBP after 10 min standing (mm Hg)	135.2 (21.4)	124.4 (16.0)	0.012^ a^	0.047
DBP after 10 min standing (mm Hg)	82.2 (11.2)	78.7 (8.8)	0.122 ^a^	0.185
Heart rate after 10 min standing (beats/min)	70.9 (10.6)	74.5 (9.9)	0.119 ^b^	0.185
Orthostatic hypotension	4 (10)	7 (17.5)	0.330 ^c^	0.385
SBP end of paced breathing (mm Hg)	146.0 (19.9)	139.1 (20.5)	0.132 ^a^	0.185
DBP end of paced breathing (mm Hg)	84.3 (9.8)	81.6 (10.6)	0.249 ^a^	0.301
Heart rate end of paced breathing (beats/min)	57.9 (6.9)	63.7 (8.1)	0.001 ^a^	0.018
Respiratory rate standing (cycles/min)	14.9 (2.7)	15.9 (3.1)	0.161 ^a^	0.201
Δ Respiratory rate (cycles/min)	0.9 (1.9)	0.9 (1.6)	0.496 ^b^	0.526
VAS stress score standing	33.7 (22.4)	20.4 (20.4)	0.007 ^b^	0.041
VAS stress score paced breathing	42.2 (23.6)	28.5 (23.3)	0.019 ^b^	0.055

Continuous variables are expressed as mean (SD), categorical variables are expressed as n (%). Significant results are shown in bold typeface. ^a^ Student's t-test; ^b^ Mann-Whitney's U-test; ^c^ Chi-squared test; ^d^ correction for multiple testing by means of the Benjamini-Hochberg procedure with a 5% False Discovery Rate (FDR). NC: normal cognition (controls); MCI: mild cognitive impairment; SBP: systolic blood pressure; DBP: diastolic blood pressure; Δ respiratory rate: respiratory rate standing- respiratory rate baseline; VAS: visual analogue scale (score range 0–100, higher scores indicate greater stress).

Table 3 displays the results of the PSA of HRV for the three main indices considered in our study: LFn, HFn and LF/HF. TP, LF and HF can be found in Table S2 and did not show statistically significant differences between the two groups (independent samples t-tests on log_10_ - transformed data, all p >0.1). Since the HRV indices are presented untransformed for descriptive purposes, please refer to Table S3 for the log _10_-transformed values.

**Table 3:** Power spectral analysis of heart rate variability in the two groups of subjects

Variable	NC (n = 40)	MCI (n = 40)	p	q b	adjusted p c
LFn (n.u)					
baseline	48.7 (18.1)	49.2 (20.0)	0.910	0.910	
standing	65.8 (17.0)	52.7 (20.0)	0.002	0.018	0.009
paced breathing	38.1 (18.0)	49.1 (18.7)	0.009	0.041	0.006
Δ standing	17.1 (16.8)	3.6 (25.7)	0.007	0.041	0.012
Δ paced breathing	−10.6 (20.7)	−0.1 (23.1)	0.036	0.092	
HFn (n.u) a					
baseline	30.9 (15.7)	27.5 (13.1)	0.430	0.470	
standing	18.5 (10.3)	26.2 (14.3)	0.015	0.049	0.018
paced breathing	47.7 (18.5)	38.6 (17.3)	0.035	0.092	
Δ standing	−12.5 (14.2)	−1.4 (13.4)	0.001	0.018	0.003
Δ paced breathing	16.7 (20.0)	11.0 (17.1)	0.374	0.422	
LF/HF a					
baseline	2.4 (2.2)	2.7 (2.7)	0.605	0.623	
standing	5.2 (3.8)	3.4 (3.3)	0.003	0.021	0.006
paced breathing	1.2 (1.5)	1.8 (1.5)	0.014	0.049	0.039
Δ standing	2.8 (2.6)	0.7 (3.0)	<0.001	0.004	<0.001
Δ paced breathing	−1.2 (2.0)	−0.9 (2.8)	0.075	0.154	

HRV indices, expressed as mean (SD), in baseline conditions and during and in response to (Δ) provocative tests (active standing, paced breathing). Significant results are shown in bold typeface.

a statistical analyses performed on log _10_ - transformed variable;

b correction for multiple testing by means of the Benjamini-Hochberg procedure with a 5% False Discovery Rate (FDR);

c adjustment for potential confounders by means of multiple linear regression (see text for details). NC: normal cognition (controls); MCI: mild cognitive impairment; n.u.: normalized units; LFn: low frequency power (normalized); HFn: high frequency power (normalized); LF/HF: LF to HF ratio; Δ standing: standing HRV index - baseline HRV index; Δ paced breathing: paced breathing HRV index - baseline HRV index.

After correction for multiple testing, there were a number of significant differences in the HRV indices between the two groups, which were confirmed after adjustment for potential confounders. Notably, significant differences in the HRV indices between the two groups were not found in baseline conditions, but only during and in response to the provocative tests. During active standing, in MCI subjects compared to controls, LFn and LF/HF were significantly lower (p = 0.018 and p = 0.021 respectively) and HFn was significantly higher (p = 0.049). In terms of the response to active standing, in MCI subjects ΔLFn and ΔLF/HF were significantly less positive (p = 0.041 and p = 0.004 respectively) and ΔHFn was significantly less negative (p = 0.018) –i.e. the physiological increase/decrease in HRV indices was significantly smaller. During paced breathing, in MCI subjects relative to controls, LFn and LF/HF were significantly higher (p = 0.041 and p = 0.049 respectively). These differences remained significant (standing: LFn p = 0.009, HFn p = 0.018, LF/HF p = 0.006; Δ standing: LFn p = 0.012, HFn p = 0.003, LF/HF p<0.001; paced breathing: LFn p = 0.006, LF/HF p = 0.039) after adjustment for potential confounders (CIRS-m and VAS-stress scores, medication use and HR; please refer to the Statistical analysis for full details)

**Figure 3:**
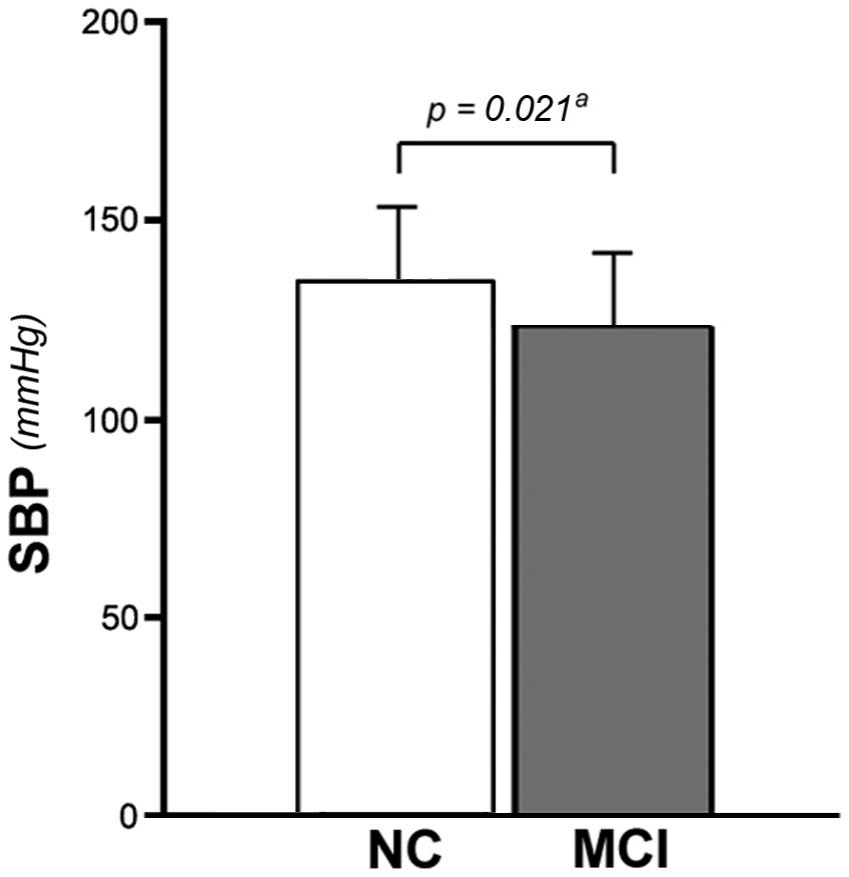
Systolic blood pressure after 10 minutes of active standing in the two groups of subjects. Bars represent the mean and error bars represent one standard deviation (SD). ^a^ adjustment for potential confounders by means of multiple linear regression (see text for details). SBP: systolic blood pressure; NC: normal cognition (controls); MCI: mild cognitive impairment.

In terms of the response to paced breathing there were no significant differences between MCI subjects and controls–i.e. the physiological decrease in LFn and LF/HF and the physiological increase in HFn were similar (p = 0.092, p = 0.154 and p = 0.422 respectively). Figure 3 illustrates that the SBP after 10 minutes of standing was still significantly lower (p = 0.021) in the MCI group following adjustment for potential confounders (CIRS-m and VAS-stress standing scores as well as antihypertensive medication use).

## Discussion

To the best of our knowledge this is the first study, based on a reliable and comprehensive assessment of autonomic function by means of PSA of HRV in different conditions, to demonstrate autonomic dysfunction in subjects with MCI compared to cognitively normal controls. In particular, there are two main findings. First, the autonomic dysfunction is not detectable in baseline conditions but becomes manifest only during and in response to the provocative tests and is consistent with an asymmetric involvement of the two branches of the ANS, i.e. a preferential impairment of the orthosympathetic component. Second, it is associated with orthostatic BP dysregulation.

The lack of significant differences in HRV indices between the two groups in baseline conditions is in line with the previous report by Zulli et al. [19], but contrasts with the results of studies in subjects with AD that show autonomic impairment already in the resting supine state [18], [19], [21]. It is therefore reasonable to suppose that MCI is characterized by a milder autonomic dysfunction that is revealed only when the ANS is challenged. The higher baseline HR observed in MCI subjects should therefore not be interpreted as an expression of dysautonomia, but is likely to be a spurious finding in the context of multiple testing, also because no differences in resting HR relative to controls have been found in MCI [19], [36] or even AD subjects [13], [15], [18], [19], [21].

As far as the provocative tests were concerned, in MCI subjects during active standing LFn and LF/HF were lower and HFn was higher, indicating a shift of sympathovagal balance towards parasympathetic modulation, whereas during paced breathing LFn and LF/HF were higher, indicating a shift of sympathovagal balance towards orthosympathetic modulation (which translated into an increased HR relative to controls at the end of this part of the experimental session).

Most important, when focusing on the Δ HRV indices – which represent the changes in HRV from baseline to stimulation and enable the crucial assessment of the dynamic range of the ANS [37], [88]– the two branches of the ANS appear to be differentially affected. In response to active standing the physiological increase in LFn and LF/HF and the physiological decrease in HFn were smaller in MCI subjects, reflecting a dysfunction of the orthosympathetic system, which is the limb of the ANS gauged by this test. This is consonant with findings in subjects with dementia (AD): Giubilei at al. found a marked blunting of the response to head-up tilt [18] and de Vilhena et al., although not directly evaluating variations in HRV indices, provide data that show unchanged LFn and HFn in the transition from the supine to the active standing position [21]. In response to paced breathing the physiological increase in HFn and the physiological decrease in LFn and LF/HF were not significantly different between the two groups, suggesting that in MCI subjects there is no dysfunction of the parasympathetic system, which is the limb of the ANS explored by this test. Since there are no studies employing paced breathing protocols and HRV analysis not only in MCI but also in dementia, these results are more difficult to place into context, but impaired clinical parasympathetic tests like the HR response to deep breathing [13] have been described in subjects with AD as well as with other forms of dementia [16]. Thus, it can be presumed that parasympathetic dysfunction sets in at a more advanced stage of cognitive decline. However, it must also be stressed that there is inconsistency in the literature as to the HR response of AD subjects to deep breathing, maybe due to methodological discrepancies across different studies, with reports of no differences relative to controls [12], [15], [16]. Therefore, an alternative hypothesis could be that there occurs no parasympathetic dysfunction in either MCI or dementia.

Whatever the case, our findings suggest that in subjects with MCI there is a preferential impairment of the orthosympathetic limb of the ANS. The pathophysiological mechanism mediating the association between MCI and such pattern of autonomic dysfunction could be right insular damage. In fact the insula is a key hub of the central autonomic network (CAN), a complex network of central nervous system units that is implicated both in cognitive functioning and in the regulation of ANS output via projections on preganglionic parasympathetic and orthosympathetic neurons of the dorsal motor nucleus of the vagus and the intermediolateral cell columns of the spinal cord [109]. It is acknowledged, based on histopathological and neuroimaging studies, that the insulae are affected in the early preclinical stages of dementia [110], [111] and there is emerging evidence that the right insula may be the most involved [112], [113]. It is generally accepted that the right insula is predominantly responsible for orthosympathetic CV control, as testified by a body of functional neuroimaging and cardiovascular data in healthy young volunteers [114], [115], as well as patients with epilepsy [116], with tinnitus [117] and, despite some amount of controversy, with stroke [116]. In addition, it has more recently been shown that in non demented older adults free of significant CV disease an absolute reduction in right insular blood flow on functional magnetic resonance imaging was significantly associated with a greater drop in SBP during a sit-stand manoeuvre [113].

It can also be speculated that the pathophysiological substrate of the attenuated orthosympathetic activation and parasympathetic withdrawal in MCI subjects during active standing is a lesion of the locus coeruleus (LC). Indeed the LC, another component of the CAN, is among the first brain sites undergoing histopathological changes in MCI [118] and is activated by BP reductions [119], producing an increase in orthosympathetic and a decrease in parasympathetic activity [120].

In subjects with MCI the SBP after 10 minutes of active standing was lower than in controls, suggesting orthostatic BP dyregulation. This corroborates results from previous scant research in the area of BP regulation in MCI, indicating a three-fold increase in the prevalence of OH in MCI compared to cognitively normal subjects [51] and a higher prevalence of MCI relative to normal cognition in subjects with abnormal nocturnal BP profiles [50]. However, unlike Mehrabian et al. [39] we found that the difference in the prevalence of OH in the two groups, although in the expected direction (i.e. prevalence of OH about 1.8-fold higher in MCI), was not statistically significant. Such discrepancy is very likely explained by the much smaller sample size of our study coupled with the fact that OH is a binary yes/no variable based on an arbitrary threshold definition and as such is less sensitive in capturing differences between the groups than is a continuous variable like standing SBP.

Orthostatic BP dysregulation could hold particular clinical significance in elderly subjects in whom a shift of the cerebral autoregulation curve towards higher BP values increases vulnerability to cerebral hypoperfusion [121]. Indeed it has been shown that even in healthy elderly without OH there was a significant decrease in cortical oxygenation during 10 minutes of active standing when compared with young subjects with the same postural BP change [122].

It could therefore be conjectured that MCI leads to dysautonomia through a disruption in central autonomic control, wherever the site of the lesion within the CAN, and that a vicious circle might thereby ensue by which dysautonomia-associated orthostatic SBP dysregulation may result in cerebral hypoperfusion and brain damage that contribute to the progression of cognitive decline towards overt dementia. This latter possibility is of considerable clinical interest in the quest for predictors of evolution to dementia and deserves some discussion. It is true that in the literature there is a lack of direct evidence that dysautonomia causes cognitive decline since the studies investigating HRV in dementia or MCI [16]–[22], [54] are cross-sectional and the prospective analyses from the study by Britton et al. [23] reveal only a weak association between HRV at baseline and changes in cognition at follow-up. However, Britton et al. focus on a population at lower risk of cognitive decline (middle-aged adults not selected for cognitive impairment) and do not carry out detailed neuropsychological testing. Also, the literature provides several distinct pieces of evidence that fit in well with the hypothesis that dysautonomia may accelerate cognitive decline via orthostatic BP dysregulation and WMLs. In a sample of geriatric patients a SBP <130 mm Hg was demonstrated to be an independent predictor of WMLs [42]. In MCI subjects a greater WML burden increased the likelihood and rate of cognitive decline [123] as well as the risk of conversion to dementia [124]. To close the circle, large prospective population-based studies in older adults have shown that a baseline SBP <130 mm Hg [125] or <110 mm Hg [126] was associated with worse cognitive performance at follow-up and that an increase in SBP decreased the risk of developing dementia in people on antihypertensive medication (per 10 mm Hg: relative risk = 0.93) [127]. Furthermore, within a group of MCI subjects, reduced HRV indices have been reported to positively correlate with WML severity [54]. Finally, cerebral hypoperfusion [128] and right insular atrophy [129] were observed on neuroimaging in converting versus stable MCI subjects.

Also, it can be supposed that in MCI subjects dysautonomia-associated orthostatic SBP dysregulation could play a role in the increased risk of falls [47], [48] and subsequent morbidity and mortality, through haemodynamic changes [41] which may be superimposed on a background of gait and balance disturbances due to WMLs [44] possibly induced by the lower SBP [42].

The ongoing follow-up of our study population will be important to better clarify the clinical relevance of autonomic dysfunction (and orthostatic BP dysregulation) in terms of the likelihood and rate of progression to dementia, the incidence of falls and the risk of mortality, be it fall-related or more generally associated with the disruption of the homeostasis-maintaining function of the ANS [11]. Along this avenue of research, which needs the contribution of further and larger studies, autonomic dysfunction could prove to be a predictor of adverse health outcomes in MCI subjects and autonomic assessment could then be used to help identify high-risk individuals to whom appropriate interventions should be targeted.

Among the strengths of our study- including fully controlled experimental conditions, the use of provocative tests to differentially challenge the two branches of the ANS and performance of PSA according to rigorous methodological standards- was the fact that we collected information on a number of potential confounders and adjusted for them when appropriate. These included drug history, comorbidity and protocol-induced emotional stress. With regard to the latter two items it is worth mentioning that both CIRS and VAS-stress scores were lower (or marginally lower) in MCI subjects than in controls. The lesser comorbidity found in subjects with MCI could stem from the nature of our sample, consisting of older adults referred to a Geriatric Unit for specialist care by their GPs. In fact it may be assumed that referral for suspected cognitive disturbances is a routine practice while geriatric evaluation for other clinical conditions is deemed necessary only when they are multiple or particularly severe. The lower levels of stress reported by MCI subjects could be attributed to different reasons. Difficulty in using the VAS scale does not appear plausible because this simple scale has been successfully employed for the rating of quality of life in individuals with mild to moderate AD [130]. A greater tendency to understate stress is also unlikely since the two groups did not differ in scores on the short form of the Marlowe-Crowne scale [131] that investigates socially desirable responding (20.1±2.8 MCI vs 19.7±2.6 NC, p = 0.539, data not shown). It therefore appears that patients with MCI actually experience less stress. Although we are not aware of studies comparing individuals with MCI and NC on their response to specific stressors, it is known that apathy is one of the most frequent behavioural symptoms in MCI [132] and emotional blunting might reduce the appraisal of stress. Also, subjects with MCI often display anosognosia for cognitive deficits [133] and in a sample of older adults spanning the cognitive spectrum it has been demostrated that anosognosia for dementia positively correlated with anosognosia for perceived stress [134].

There are some limitations to our study. Its cross-sectional design precludes conclusions on the direction of causality in the association between MCI and autonomic dysfunction, which can only be elucidated by longitudinal studies. As previously mentioned, the follow-up of the study population will also be particularly important to better clarify the prognostic significance of autonomic dysfunction (and orthostatic BP dysregulation) in terms of adverse health outcomes, and is currently under way.

Medication use by the study participants is a critical issue which requires discussion. Since this is an exploratory study of a clinical population – and specifically of older adults, which implies a particularly high rate of comorbidity-related polypharmacy – the inclusion of medication users is consonant with the nature of the study. In fact, given that many drugs have a presumed impact on the ANS, the approach of excluding all medications that may affect HRV (e.g. [18], [21], [22], would have yielded a sample of subjects not representative of the underlying population. On the other hand, including all medications (e.g. [36], [54]) would have resulted in an undue pharmacological influence on HRV. Thus, we chose to exclude some medications while accepting others (please see Methods for the exclusion/inclusion criteria), in line with previous studies on HRV involving subjects likely to be on polypharmacy because of general age-related comorbidity (e.g. [19], [135]–[139]) or specific clinical conditions like heart disease (e.g. [137], [140], [141]). Although the allowed medications (ACE-Is/ARBs, diuretics, peripherally-acting CCBs, SSRIs, benzodiazepines) can be considered a source of confounding, and although a subgroup analysis excluding them is not feasible on account of the marked decrease in sample size (n = 12 NC vs n = 10 MCI) and hence in statistical power, it should be remarked that there was no significant difference between the two groups in the prevalence of use of each of these classes of medications, enabling meaningful comparisons to be made. Moreover, the statistical analyses were also corrected for medication use. Therefore it seems reasonable to suppose that medication use is not inappropriately interfering with our findings. This line of reasoning holds true for all the medications mentioned but could be of even more relevance in the case of psychotropic medications with a potential to affect both HRV and cognition. Indeed, as far as benzodiazepines were concerned, since they can be taken not only regularly but also as needed, in order to ensure that benzodiazepine use was predictable, thereby enabling comparison between groups, only regular users were allowed while intermittent users were asked to refrain from benzodiazepine consumption prior to testing (please see Methods and Table 1).

As to the medication selection criteria, even if we acknowledge there may be some degree of arbitrariness in our choice of exclusion/inclusion criteria it is worth noting that we strived to make it as objective as possible by grounding it in the literature. To this end we decided to exclude medications which have been consistently and significantly shown to affect HRV: beta-blockers [8], [142], alpha-blockers [143], [144]), centrally-acting CCBs [145]–[150]), class I and class III antiarrhytmic drugs [8], [142], digoxin [142], tryciclic antidepressants [57], [151], [152], antipsychotics [153]–[155], SNRIs [56], [152] and cholinesterase inhibitors [18], [156]. Atypical antidepressants (e.g. mirtazapine, trazodone) were also excluded because very few studies exist on their effect on HRV and results are conflicting [57], [151], [157]–[159]. We instead allowed medications whose influence on HRV has consistently been found to be nil to limited – selective serotonin reuptake inhibitors (SSRIs) [56], [57], [151], [152] and diuretics [160], [161] - or remains controversial despite a rather large body of research- peripherally-acting CCBs [148], [149], [161]–[164] and benzodiazepines [154], .

Although benzodiazepines have the potential to affect the activity of the central ANS, since it contains GABAergic neurons [170], their impact on HRV can be considered at least controversial. In fact the oral benzodiazepines, used for the treatment of insomnia and anxiety in community-dwelling subjects, unlike the intravenous ones [171], [172] have mostly been reported to have no effect on HRV [165], [167], [169], with some exception [166], [168]. Moreover, a recent and larger study in patients with schizophrenia [154], making no distinction between routes of administration, demonstrated that daily benzodiazepine dose was not associated with frequency-domain measures of HRV.

Finally, ACE-inhibitors (ACE-Is)/angiotensin II receptor blockers (ARBs) were also permitted. Despite the fact that the literature is largely in favour of an effect of these medications on HRV [142], [163], [173], [174], with only some occasional evidence of no effect [160]–[162], we still believed it would be reasonable to allow them based on the notion that hypertension has a very high prevalence in the elderly [175] and that they are the most commonly prescribed antihypertensives [176], meaning that their exclusion would greatly detract from the generalizability of our findings.

Since the exclusion/inclusion criteria include several cardioactive medications often employed as antihypertensives, their application could also be having an impact on the hypertensive subjects in the study population. With regard to the exclusion criteria, excluding some antihypertensive medications could mean selecting untreated (i.e. unrecognized) hypertensives as well as hypertensives treated with a lesser number of medications- uncontrolled and potentially uncontrolled hypertensives respectively. Although such possibility cannot be definitely ruled out because the study, with a single baseline BP measurement and no 24-hour ambulatory BP monitoring [177], was not designed to diagnose hypertension or to assess its pharmacological control, two points are worth making. First, if there actually are unrecognized hypertensives we would not expect them to be differently distributed within the two groups. Moreover, the 56% prevalence of hypertension found in the study population is very close to the 66% reported when investigating an ambulatory elderly population [178], considering that in our case some of the hypertensives were lost due to exclusion criteria; this seems to suggests that unrecognized hypertension is not a major problem in our study, consistently with the fact that it enrolled older adults accessing secondary care who are likely to have been screened for high BP by their GPs. Second, hypertensives treated with a lesser number of medications are not necessarily uncontrolled hypertensives; on the contrary they may be subjects whose hypertension is more amenable to therapy. With regard to the inclusion criteria, allowing medications with either consistent (e.g. ACE-Is/ARBs) or controversial (e.g. peripherally-acting CCBs) evidence of an effect on HRV could also be a methodological concern. In fact, while it is recognized that untreated hypertension is characterized by dysautonomia [9] the autonomic profile of treated hypertensives appears to depend on the medication used, beyond its BP-lowering effect (e.g. [148]–[150], [160], [163], [174]). Therefore, it could be objected that, although all subjects recorded as hypertensives in our study were known hypertensives on stable treatment and there was no significant difference in the prevalence of hypertension between the two groups, their autonomic status could differ. However, this does not seem to be the case because, as already discussed, medication use was similar in MCI and NC subjects and it was also adjusted for in statistical analyses.

As to the question of how representative the study sample is of a clinical and/or community-dwelling elderly population, some issues should be borne in mind. The use of exclusion criteria inevitably implies that the subjects enrolled cannot be fully representative of the population considered for inclusion. In fact, even though we limited exclusion criteria as much as possible, the 80 subjects included differed from the overall 475 in terms of several characteristics. On account of the exclusion of a number of medications and clinical conditions (including dementia), they showed less comorbidity and polypharmacy as well as better functional and cognitive status, as expected. The borderline higher education could be a consequence of the exclusion of individuals suffering from dementia since there is a recognized inverse relationship between education and risk of dementia [179]. The female predominance might stem from the higher prevalence among males of excluded CV diseases such as CAD and HF [180]. The lower prevalence of diuretic use can speculatively be ascribed to the fact that diuretics are mainstay of HF treatment in the elderly [181] and HF was excluded. Furthermore, the generalizability of our findings to the older community-dwelling population at large may be limited by the nature of the recruited population (i.e. outpatients attending a Geriatric Unit) which may have biased the sample towards subjects with poorer cognitive status and worse health. However, with regard to cognitive status, although some degree of bias cannot be excluded, it is important to note that our Geriatric Unit does not exclusively address cognitive disturbances but a wide variety of age-related health problems and that in our experimental design subjects were invited to undergo on-site neuropsychological testing independently of cognitive functioning and responded with a very high participation rate (113 out of 117, i.e. about 97%, agreed to testing). Also, as far as overall health was concerned, selection bias probably operated in two opposite directions, with outpatients referred to the Geriatric Unit having worse health but then being selected for better health based on the exclusion criteria.

The sample size was relatively small and, although in line with or indeed larger than that of other HRV studies on subjects with MCI and AD [18], [19], [21], [54] it did not allow us to investigate autonomic function across different MCI subtypes [1].

The diagnosis of MCI was based on current consensus criteria: objective cognitive impairment documented by neuropsychological testing, essentially preserved functional independence and absence of dementia. Consistently with most other studies [75] we did not take into account the subjective and longitudinal criteria. The first is defined by a cognitive concern reported by the patient and/or an informant and whether it should be adopted is a matter of debate [75], [182]. In fact patients with MCI often lack self-awareness [75], [133], [183] while subjects who complain about cognition may be depressed or anxious [75], [184]. Although informant reports have generally been shown to correlate well with objective cognitive performance in MCI, their clinical use is hampered by the lack of validated rating instruments and the potential bias stemming from the informant's personal characteristics [183] including the fact that MCI caregivers are mostly spouses [185] and thus older adults who may themselves be cognitively impaired. The second criterion corresponds to an objective cognitive decline over time and its omission is due to the cross-sectional design of the study. Prospective neuropsychological testing could probably have improved the accuracy of MCI classification by providing evidence of intraindividual change in cognition. However, recent guidelines recognize that an MCI diagnosis will likely need to be given without the benefit of serial cognitive assessments [60]. Also, it should be noted that whereas declining cognition is suggestive of MCI, the reverse may not be true- i.e. a significant proportion of MCI patients actually remain stable at follow-up [186].

Tidal volume was not controlled or measured, but subjects were simply asked to breathe normally during baseline conditions and active standing and at a “comfortable” tidal volume during paced breathing. Although this is a potential methodological issue, it is bound to have little practical meaning since there is no reason to assume that tidal volume was different in the two groups and it is acknowledged that, compared to respiratory rate (which was indeed taken into account) tidal volume is a much less potent determinant of HF [92] with negligible effects within the physiological breathing range [93]. Also, controlling or measuring tidal volume would have increased the stress and complexity of the protocol. On such grounds, in our study, like in the vast majority of HRV publications [93], tidal volume was not considered.

Lastly, it can also be argued that the presence of subjects with bradycardia may be affecting our findings by reducing the number of beats available for analysis. However, ectopics were edited by linear interpolation rather than simple deletion (please see HRV analysis), implying no loss of data from the 5-minute recordings, and it has been reported that PSA on data segments 3 to 5 minutes long provides reliable results, regardless of whether the subject has a high or low basal HR [187]. In addition, corrections for HR were performed in the statistical analyses.

## Conclusions

To the best of our knowledge this is the first study, based on a reliable and comprehensive assessment of autonomic function by means of PSA of HRV in different conditions, to demonstrate autonomic dysfunction in subjects with MCI compared to cognitively normal controls, after adjustment for potential confounders. The autonomic dysfunction was not detectable in baseline conditions but only during and in response to provocative tests, was consistent with orthosympathetic impairment, and was associated with orthostatic BP dysregulation. The underlying physiopathological mechanism could be disruption of central autonomic control due to right insular or locus coeruleus damage. The potential clinical relevance of autonomic dysfunction in MCI is manifold, as there is convincing evidence it may play a role in progression to dementia, in the increased risk of mortality and falls, and it may be affected by pharmacological treatments. Longitudinal studies will be required to assess the direction of causality and to better clarify the prognostic significance of the autonomic dysfunction in terms of adverse health outcomes.

## Supporting Information

Table S1 **Neuropsychological test scores by cognitive domain in the two groups of subjects.**
(DOCX)

Table S2 **Total power and absolute low- and high-frequency powers in the two groups of subjects.**
(DOCX)

Table S3 **Log _10_ – transformed HRV indices in the two groups of subjects.**
(DOCX)
