# Correction: Autonomic Dysfunction in Mild Cognitive Impairment: Evidence from Power Spectral Analysis of Heart Rate Variability in a Cross-Sectional Case-Control Study

**DOI:** 10.1371/journal.pone.0109231

**Published:** 2014-09-19

**Authors:** The PLOS ONE Staff

There are errors in Tables 1, 2 and 3. In these tables, significant results (p<0.05) should be in bold typeface. Please see the corrected tables here.

**Table 1:** Baseline characteristics of the study subjects.

Variable	NC (n = 40)	MCI (n = 40)	p
Age (years)	77.8 (4.5)	79.4 (5.3)	0.145 b
Gender, female	33 (82.5)	27 (67.5)	0.121 d
Education (years)	11.4 (4.4)	10.5 (4.4)	0.279 c
BMI (kg/m^2^)	24.1 (3.0)	24.6 (3.2)	0.431 b
Hypertension	21 (52.5)	24 (60.0)	0.499 d
SBP (mm Hg)	134.8 (19.2)	129.0 (17.4)	0.158 b
DBP (mm Hg)	75.2 (8.7)	73.6 (8.3)	0.404 b
Heart rate (beats/min)	65.2 (8.7)	69.6 (9.6)	**0.035** b
Respiratory rate (cycles/min)	14.0 (2.4)	15.0 (2.8)	0.104 b
Smoking	2 (5.0)	1 (2.5)	1.000 e
Alcohol (AU/day)	1.3 (1.6)	1.3 (1.5)	0.968 c
Coffee (cups/day)	1.5 (1.0)	1.3 (1.1)	0.317 c
Physical activity (MET-hours/week)	68.4 (39.3)	57.6 (39.8)	0.144 c
Family history of premature CAD	7 (17.5)	7 (17.5)	1.000 d
Glucose (mg/dl)	87.7 (9.8)	91.5 (10.0)	0.107 b
Total cholesterol (mg/dl)	222.9 (37.8)	227.0 (33.9)	0.635 b
LDL cholesterol (mg/dl)	137.6 (33.0)	139.7 (28.0)	0.768 b
HDL cholesterol (mg/dl)	69.2 (20.5)	65.8 (18.2)	0.470 c
Triglycerides (mg/dl)	104.0 (32.4)	106.7 (36.4)	0.745 b
Target organ damage			
LVH-echocardiography	16 (40.0)	20 (50.0)	0.369 d
Carotid atherosclerosis			
Thickening (IMT>0.9 mm)	35 (87.5)	30 (75.0)	0.152 d
plaque/s	33 (82.5)	29 (72.5)	0.284 d
Number of medications	3.3 (1.7)	3.8 (2.2)	0.311 b
Antihypertensive medications			
ACE-Is/ARBs	16 (40.0)	23 (57.5)	0.117 d
Diuretics	7 (17.5)	9 (22.5)	0.576 d
CCBs (peripherally-acting)	3 (7.5)	8 (20.0)	0.105 d
Psychotropic medications			
SSRIs	9 (22.5)	13 (32.5)	0.317 d
Benzodiazepines a	8 (20.0)	7 (17.5)	0.775 d
BADL score	5.5 (0.5)	5.6 (0.6)	0.325 c
IADL score	7.4 (1.2)	6.7 (1.6)	**0.006** c
MMSE score	28.6 (1.0)	26.8 (2.0)	**<0.001** b
CIRS-s score	1.6 (0.2)	1.4 (0.2)	**0.029** b
CIRS-m score	2.5 (1.3)	1.7 (1.1)	**0.006** c
STPI-T score	19.5 (5.7)	19.6 (5.7)	0.843 c
GDS-s score	3.4 (3.1)	3.2 (2.8)	0.747 c

Continuous variables are expressed as mean (SD), categorical variables are expressed as n (%). Significant results are shown in bold typeface.

a Refers to regular use. Intermittent users (n = 2 in each group) were asked to refrain from use in the two days prior to testing;

b Student’s t-test;

c Mann-Whitney’s U-test;

d Chi-squared test;

e Fisher's exact test. NC: normal cognition (controls);

MCI: mild cognitive impairment; BMI: body mass index; SBP: systolic blood pressure; DBP: diastolic blood pressure; AU: alcohol units (1 AU = 10 g of alcohol); MET: metabolic equivalent (energy expenditure index, 1 MET =  1 kcal•kg^-1^•h^-1^); CAD: coronary artery disease; LDL: low density lipoprotein; HDL: high density lipoprotein; LVH: left ventricular hypertrophy; IMT: intima-media thickness; ACE-Is: angiotensin converting enzyme inhibitors; ARBs: angiotensin II receptor blockers; CCBs: calcium-channel blockers; SSRIs: selective serotonin reuptake inhibitors; BADL: basic activities of daily living (score range 0-6, higher scores indicate greater functional independence); IADL: instrumental activities of daily living (score range 0-8, higher scores indicate greater functional independence); MMSE: mini mental state examination (score range 0-30, higher scores indicate better cognitive function); CIRS-s: cumulative illness rating scale severity (score range 1-5, higher scores indicate greater comorbidity); CIRS-m: cumulative illness rating scale morbidity (score range 0-13, higher scores indicate more severe comorbidity); STPI-T: state trait personality inventory- trait anxiety subscale (score range 10-40, higher scores indicate greater trait anxiety); GDS-s: geriatric depression scale short form (score range 0-15, higher scores indicate greater depressive symptoms).

**Table 2:** Characteristics of the study subjects during the experimental session.

Variable	NC (n = 40)	MCI (n = 40)	p	q d
SBP after 1 min standing (mm Hg)	134.7 (20.3)	127.2 (18.0)	0.088 a	0.157
DBP after 1 min standing (mm Hg)	82.1 (10.0)	78.1 (10.1)	0.080 a	0.156
Heart rate after 1 min standing (beats/min)	71.6 (11.0)	75.7 (11.3)	0.115 b	0.185
SBP after 3 min standing (mm Hg)	137.7 (18.9)	129.0 (16.7)	**0.034** a	0.092
DBP after 3 min standing (mm Hg)	82.1 (10.4)	78.7 (8.5)	0.111 a	0.185
Heart rate after 3 min standing (beats/min)	68.8 (9.6)	73.3 (10.0)	**0.047** a	0.103
SBP after 5 min standing (mm Hg)	134.8 (20.5)	128.1 (16.6)	0.115 a	0.185
DBP after 5 min standing (mm Hg)	81.7 (10.1)	78.6 (9.0)	0.150 a	0.194
Heart rate after 5 min standing (beats/min)	69.5 (11.3)	73.6 (9.8)	0.085 a	0.157
SBP after 10 min standing (mm Hg)	135.2 (21.4)	124.4 (16.0)	**0.012** a	**0.047**
DBP after 10 min standing (mm Hg)	82.2 (11.2)	78.7 (8.8)	**0.122** a	0.185
Heart rate after 10 min standing (beats/min)	70.9 (10.6)	74.5 (9.9)	0.119 b	0.185
Orthostatic hypotension	4 (10)	7 (17.5)	0.330 c	0.385
SBP end of paced breathing (mm Hg)	146.0 (19.9)	139.1 (20.5)	0.132 a	0.185
DBP end of paced breathing (mm Hg)	84.3 (9.8)	81.6 (10.6)	0.249 a	0.301
Heart rate end of paced breathing (beats/min)	57.9 (6.9)	63.7 (8.1)	**0.001** a	**0.018**
Respiratory rate standing (cycles/min)	14.9 (2.7)	15.9 (3.1)	0.161 a	0.201
Δ Respiratory rate (cycles/min)	0.9 (1.9)	0.9 (1.6)	0.496 b	0.526
VAS stress score standing	33.7 (22.4)	20.4 (20.4)	**0.007** b	**0.041**
VAS stress score paced breathing	42.2 (23.6)	28.5 (23.3)	**0.019** b	0.055

Continuous variables are expressed as mean (SD), categorical variables are expressed as n (%). Significant results are shown in bold typeface.

a Student’s t-test;

b Mann-Whitney’s U-test;

c Chi-squared test;

d correction for multiple testing by means of the Benjamini-Hochberg procedure with a 5% False Discovery Rate (FDR).

NC: normal cognition (controls); MCI: mild cognitive impairment; SBP: systolic blood pressure; DBP: diastolic blood pressure; Δ respiratory rate: respiratory rate standing- respiratory rate baseline; VAS: visual analogue scale (score range 0-100, higher scores indicate greater stress).

**Table 3:** Power spectral analysis of heart rate variability in the two groups of subjects.

Variable	NC (n = 40)	MCI (n = 40)	p	q b	adjusted p c
LFn (n.u)					
baseline	48.7 (18.1)	49.2 (20.0)	0.910	0.910	
standing	65.8 (17.0)	52.7 (20.0)	**0.002**	**0.018**	**0.009**
paced breathing	38.1 (18.0)	49.1 (18.7)	**0.009**	**0.041**	**0.006**
Δ standing	17.1 (16.8)	3.6 (25.7)	**0.007**	**0.041**	**0.012**
Δ paced breathing	-10.6 (20.7)	-0.1 (23.1)	**0.036**	0.092	
HFn (n.u) a					
baseline	30.9 (15.7)	27.5 (13.1)	0.430	0.470	
standing	18.5 (10.3)	26.2 (14.3)	**0.015**	**0.049**	**0.018**
paced breathing	47.7 (18.5)	38.6 (17.3)	**0.035**	**0.092**	
Δ standing	-12.5 (14.2)	-1.4 (13.4)	**0.001**	**0.018**	**0.003**
Δ paced breathing	16.7 (20.0)	11.0 (17.1)	0.374	0.422	
LF/HF a					
baseline	2.4 (2.2)	2.7 (2.7)	0.605	0.623	
standing	5.2 (3.8)	3.4 (3.3)	**0.003**	**0.021**	**0.006**
paced breathing	1.2 (1.5)	1.8 (1.5)	**0.014**	**0.049**	**0.039**
Δ standing	2.8 (2.6)	0.7 (3.0)	**<0.001**	**0.004**	**<0.001**
Δ paced breathing	-1.2 (2.0)	-0.9 (2.8)	0.075	0.154	

HRV indices, expressed as mean (SD), in baseline conditions and during and in response to (Δ) provocative tests (active standing, paced breathing). Significant results are shown in bold typeface.

a statistical analyses performed on log _10_ - transformed variable;

b correction for multiple testing by means of the Benjamini-Hochberg procedure with a 5% False Discovery Rate (FDR);

c adjustment for potential confounders by means of multiple linear regression (see text for details).

NC: normal cognition (controls); MCI: mild cognitive impairment; n.u.: normalized units; LFn: low frequency power (normalized); HFn: high frequency power (normalized); LF/HF: LF to HF ratio; Δ standing: standing HRV index - baseline HRV index; Δ paced breathing: paced breathing HRV index - baseline HRV index.
