# Supplementary material for: Autonomic Dysfunction in Mild Cognitive Impairment: Evidence from Power Spectral Analysis of Heart Rate Variability in a Cross-Sectional Case-Control Study
Source: PLoS One. 2014 May 6;9(5):e96656. doi: 10.1371/journal.pone.0096656 (PMC4011966; doi:10.1371/journal.pone.0096656)
Supplement: Table S1 — Neuropsychological test scores by cognitive domain in the two groups of subjects. (DOCX) [file pone.0096656.s001.docx]

**Table S1.**

**Neuropsychological test scores by cognitive domain in the two groups of subjects**

| **Test** | **NC (n=40)** | **MCI (n=40)** | **P** |
| --- | --- | --- | --- |
| Attention |  |  |  |
| Digit cancellation test *(<37)* ^a,c^ | 53.5 (4.9) | 50.5 (6.0) | **0.015** ^f^ |
| Bell test *(<33)* ^a^ | 34.4 (0.8) | 31.5 (2.8) | **<0.001** ^g^ |
| Memory |  |  |  |
| Prose recall *(<7.5)* ^a,d^ | 13.1 (2.3) | 7.7 (4.7) | **<0.001** ^g^ |
| Rey-Osterrieth complex figure - delayed recall (<11.23) ^a,c^ | 22.9 (5.4) | 13.8 (6.6) | **<0.001** ^f^ |
| Digit span forward *(<4.25)* ^a,d^ | 5.7 (0.9) | 5.3 (0.8) | **0.035** ^g^ |
| Executive functions |  |  |  |
| Digit span backward *(<3.29)* ^a,d^ | 4.4 (0.6) | 3.7 (0.7) | **<0.001** ^f^ |
| Trail making test A *(≥69)* ^b,d^ | 30.1 (13.7) | 45.1 (23.7) | **0.004** ^g^ |
| Trail making test B *(≥178)* ^b,d^ | 61.7 (36.0) | 196.7 (134.1) | **<0.001** ^g^ |
| Weigl's colour-form sorting test *(<7)* ^a,d^ | 12.1 (1.8) | 9.3 (2.6) | **<0.001** ^f^ |
| Cognitive estimates - total *(>16)* ^b,e^ | 10.5 (1.6) | 13.3 (2.4) | **<0.001** ^g^ |
| Cognitive estimates - bizarre *(>4)* ^b^ | 1.7 (0.9) | 2.6 (1.1) | **<0.001** ^g^ |
| Raven's coloured progressive matrices *(<23.5)* ^a,d^ | 32.7 (4.0) | 27.3 (4.7) | **<0.001** ^g^ |
| Letter fluency *(<23)* ^a,d^ | 37.3 (7.9) | 30.4 (8.3) | **<0.001** ^f^ |
| Language |  |  |  |
| Category fluency S *(<11.25)* ^a,d^ | 18.6 (3.4) | 13.5 (3.0) | **<0.001** ^g^ |
| Picture naming *(<70)* ^a^ | 74.9 (2.8) | 69.9 (5.6) | **<0.001** ^g^ |
| Token test *(<29.25)* ^a,d^ | 33.6 (1.2) | 30.6 (2.1) | **<0.001** ^f^ |
| Visuospatial skills |  |  |  |
| Rey-Osterrieth complex figure - copy *(<30.05)* ^a,d^ | 34.8 (3.6) | 32.4 (4.1) | **0.002** ^g^ |
| Copy of geometric figures *(<10)*^a,d^ | 13.7 (0.5) | 12.5 (1.3) | **<0.001** ^g^ |
| Ideomotor praxis |  |  |  |
| De Renzi's test - right upper limb *(<65)* ^a^ | 71.6 (0.8) | 70.6 (1.7) | **0.006** ^g^ |
| De Renzi's test - left upper limb *(<65)* ^a^ | 71.0 (1.6) | 70.0 (1.8) | **0.004** ^g^ |

**Legend**

Neuropsychological test scores, expressed as mean (SD), across different cognitive domains. The italic numbers in parentheses indicate the cut-off values below or above which the test performance was considered to be abnormal (i.e. in the “worst” 10% of the published normative score distribution). Significant results are shown in bold typeface. ^a^ Higher scores indicate better cognitive performance; ^b^ Lower scores indicate better cognitive performance. ^c^ Corrected for age, gender and education; ^d^ corrected for age and education; ^e^ corrected for gender (all corrections based on published normative data). ^f^ Student's t-test; ^g^ Mann-Whitney's U-test. NC: normal cognition (controls); MCI: mild cognitive impairment.
