# Supplementary material for: Autonomic Dysfunction in Mild Cognitive Impairment: Evidence from Power Spectral Analysis of Heart Rate Variability in a Cross-Sectional Case-Control Study
Source: PLoS One. 2014 May 6;9(5):e96656. doi: 10.1371/journal.pone.0096656 (PMC4011966; doi:10.1371/journal.pone.0096656)
Supplement: Table S2 — Total power and absolute low- and high-frequency powers in the two groups of subjects. (DOCX) [file pone.0096656.s002.docx]

**Table S2.**

**Total power and absolute low- and high-frequency powers in the two groups of subjects**

| **Variable** | **NC (n=40)** | **MCI (n=40)** | **P** ^a^ |
| --- | --- | --- | --- |
| TP (ms^2^) |  |  |  |
| baseline | 1653.4 (3964.1) | 1431.0 (1395.9) | 0.678 |
| standing | 1328.3 (2419.1) | 988.5 (769.3) | 0.855 |
| paced breathing | 1968.8 (2379.4) | 1999.2 (1902.8) | 0.473 |
| ∆ standing | -325.1 (3273.8) | -442.5 (1419.3) | 0.842 |
| ∆ paced breathing | 315.4 (3184.9) | 568.3 (1813.3) | 0.732 |
| LF (ms^2^) |  |  |  |
| baseline | 404.1 (929.4) | 297.2 (367.3) | 0.706 |
| standing | 258.7 (573.2) | 176.3 (196.5) | 0.559 |
| paced breathing | 582.9 (2448.9) | 484.2 (851.4) | 0.121 |
| ∆ standing | -145.4 (464.9) | -121.0 (390.1) | 0.356 |
| ∆ paced breathing | 178.8 (1632.0) | 186.9 (579.2) | 0.146 |
| HF (ms^2^) |  |  |  |
| baseline | 222.6 (438.3) | 159.3 (174.7) | 0.921 |
| standing | 75.6 (130.8) | 127.7 (189.1) | 0.143 |
| paced breathing | 318.6 (595.7) | 320.6 (351.7) | 0.972 |
| ∆ standing | -147.0 (391.3) | -31.6 (197.8) | 0.111 |
| ∆ paced breathing | 96.0 (311.1) | 161.3 (280.0) | 0.946 |

**Legend**

HRV indices, expressed as mean (SD), in baseline conditions and during and in response to (∆) provocative tests (active standing, paced breathing). NC: normal cognition (controls); MCI: mild cognitive impairment; TP: total power; LF: low frequency power (absolute); HF: high frequency power (absolute); ∆ standing: standing HRV index - baseline HRV index; ∆ paced breathing: paced breathing HRV index - baseline HRV index.

^a^ Independent samples t-test performed on log_10_-transformed variable.
