# Supplementary material for: Autonomic Dysfunction in Mild Cognitive Impairment: Evidence from Power Spectral Analysis of Heart Rate Variability in a Cross-Sectional Case-Control Study
Source: PLoS One. 2014 May 6;9(5):e96656. doi: 10.1371/journal.pone.0096656 (PMC4011966; doi:10.1371/journal.pone.0096656)
Supplement: Table S3 — Log 10 – transformed HRV indices in the two groups of subjects. (DOCX) [file pone.0096656.s003.docx]

**Table S3.**

**Log _10_ - transformed HRV indices in the two groups of subjects**

| **Variable** | **NC (n=40)** | **MCI (n=40)** |
| --- | --- | --- |
| lg HFn (n.u.) |  |  |
| baseline | 1.42 (0.26) | 1.38 (0.25) |
| standing | 1.20 (0.25) | 1.34 (0.27) |
| paced breathing | 1.64 (0.20) | 1.54 (0.20) |
| ∆ standing | -0.23 (0.26) | -0.04 (0.22) |
| ∆ paced breathing | 0.21 (0.27) | 0.16 (0.26) |
| lg LF/HF |  |  |
| baseline | 0.23 (0.37) | 0.27 (0.39) |
| standing | 0.60 (0.32) | 0.34 (0.42) |
| paced breathing | -0.12 (0.42) | 0.11 (0.39) |
| ∆ standing | 0.38 (0.27) | 0.07 (0.39) |
| ∆ paced breathing | -0.34 (0.46) | -0.16 (0.46) |
| lg TP (ms^2^) |  |  |
| baseline | 2.94 (0.42) | 2.98 (0.40) |
| standing | 2.83 (0.47) | 2.84 (0.41) |
| paced breathing | 3.04 (0.47) | 3.11 (0.42) |
| ∆ standing | -0.11 (0.39) | -0.13 (0.50) |
| ∆ paced breathing | 0.10 (0.47) | 0.14 (0.42) |
| lg LF (ms^2^) |  |  |
| baseline | 2.21 (0.52) | 2.25 (0.44) |
| standing | 2.10 (0.46) | 2.04 (0.44) |
| paced breathing | 2.13 (0.57) | 2.33 (0.55) |
| ∆ standing | -0.11 (0.41) | -0.21 (0.54) |
| ∆ paced breathing | -0.08 (0.44) | 0.08 (0.50) |
| lg HF (ms^2^) |  |  |
| baseline | 1.98 (0.55) | 2.00 (0.46) |
| standing | 1.51 (0.54) | 1.70 (0.61) |
| paced breathing | 2.22 (0.50) | 2.23 (0.54) |
| ∆ standing | -0.47 (0.46) | -0.29 (0.53) |
| ∆ paced breathing | 0.24 (0.42) | 0.23 (0.49) |

**Legend**

Log_10_ - transformed (lg) HRV indices, expressed as mean (SD), in baseline conditions and during and in response to (∆) provocative tests (active standing, paced breathing). NC: normal cognition (controls); MCI: mild cognitive impairment; n.u.: normalized units; HFn: high frequency power (normalized); LF/HF: LF to HF ratio; TP: total power; LF: low frequency power (absolute); HF: high frequency power (absolute); ∆ standing: standing HRV index - baseline HRV index; ∆ paced breathing: paced breathing HRV index - baseline HRV index.
